# Physical activity is reduced prior to ventricular arrhythmias in patients with a wearable cardioverter defibrillator

**DOI:** 10.1002/clc.23288

**Published:** 2019-11-11

**Authors:** Ashley E. Burch, Benjamin D'Souza, J. Rod Gimbel, Ursula Rohrer, Tsuyoshi Masuda, Samuel Sears, Daniel Scherr

**Affiliations:** ^1^ East Carolina Heart Institute Greenville North Carolina; ^2^ University of Pennsylvania Philadelphia Pennsylvania; ^3^ Cardiac Rhythm Specialists Bayside Wisconsin; ^4^ Department of Medicine, Division of Cardiology Medical University of Graz Graz Austria; ^5^ ZOLL Medical Pittsburgh Pennsylvania; ^6^ East Carolina University Greenville North Carolina

**Keywords:** activity, ventricular arrhythmia, wearable cardioverter defibrillator

## Abstract

**Introduction:**

The utility of accelerometer‐based activity data to identify patients at risk of sustained ventricular tachycardia (VT) or ventricular fibrillation (VF) has not previously been investigated. The aim of the current study was to determine whether physical activity is associated with manifesting spontaneous sustained VT/VF requiring emergent defibrillation in patients with an ejection fraction of ≤35%.

**Methods:**

Patients consecutively prescribed a wearable cardioverter defibrillator (WCD) from April 2015 to May 2018 were included. Shock data and 4 weeks of physical activity data, beginning with the first week of WCD wear, were analyzed.

**Results:**

Based on the ROC curve outcome generated from 4057 patients, average daily step count during the first week accurately predicted those patients with sustained VT/VF compared to those without (shocked (n = 81) vs nonshocked (n = 3976) area under the curve, c‐index = 0.71, 95% CI = 0.65‐0.77, *P* < .001). An average cutoff of 3637 daily steps during week 1 separated the groups. Patients who averaged fewer than 3637 steps per day during the first week of WCD use were 4.3 times more likely to experience a shock than those who walked more than 3637 steps per day (OR = 4.29, 95% CI = 2.58‐7.15, *P* < .001).

**Discussion:**

Average daily step counts are lower in WCD patients who manifest spontaneous VT/VF. Whether these findings represent a causal or correlational relationship, future studies to encourage a minimum daily step count in high‐risk patients may impact the incidence of sustained VT/VF.

## 1 INTRODUCTION

Physical activity has prognostic value for cardiac patients. Following a myocardial infarction, patients self‐reported activity is a significant predictor of death.^1^, ^2^, ^3^ More objective measures of physical activity, such as walking speed or results from the 6‐minute walk test, are also predictive of clinical outcomes for cardiac patients.^4^, ^5^ Many modern cardiac devices contain accelerometers, allowing for continuous monitoring of activity. In patients with heart failure, activity data collected from these devices has been used to predict atrial arrhythmias, improved ejection fraction, heart failure hospitalization, and death.^5^, ^6^, ^7^, ^8^, ^9^, ^10^

The precision of accelerometer data has allowed investigators to calculate specific cutoff values of activity associated with adverse cardiac outcomes. A retrospective study of patients with implantable cardioverter defibrillators (ICDs) found that decreased physical activity in the months following implantation is associated with an increase in heart failure hospitalization and mortality.^6^ For every 10 minutes per day of additional time spent active, there was a 4% risk reduction in a composite variable of hospitalization and death. Zhao and colleagues (2017) analyzed activity data from 845 patients with ICDs to determine whether physical activity could predict cardiac mortality. A cutoff value of 113 minutes of activity per day was found to successfully predict cardiac death. In a recent study, accelerometer‐based activity was associated with the risk of atrial arrhythmias.^9^ Patients who were active for less than 3.5 hours per day were at a higher risk for subclinical high‐rate atrial fibrillation lasting for 6 minutes or longer. These studies demonstrate the utility of physical activity as a mechanism to identify patients at risk for cardiac events.

Ventricular arrhythmias are the leading cause of sudden cardiac death among patients with heart failure. In the months following a diagnosis of heart failure with reduced ejection fraction, patients are at an increased risk of arrhythmic death.^11^, ^12^ The wearable cardioverter defibrillator (WCD) is able to detect ventricular tachycardia (VT) and ventricular fibrillation (VF) and deliver a shock to restore sinus rhythm (Chung, 2014). Like the ICD, the WCD contains an accelerometer capable of continuous activity monitoring. Among patients with an ICD, self‐reported physical exertion is associated with an increased risk of sustained VT.^13^ However, when accounting for general fitness level, the relationship between sustained ventricular arrhythmia and physical exertion is strongest among patients who reported generally lower fitness levels or predominately sedentary behavior over the 6 months preceding an appropriate shock.^14^ The utility of an accelerometer to identify patients at risk of sustained VT/VF based on physical activity has not been investigated. The aim of the current study was to determine whether physical activity is associated with sustained VT/VF requiring emergent defibrillation in patients with an ejection fraction of ≤35%. Specifically, differences in daily step count were compared for WCD patients who received a shock for sustained VT/VF and those who did not.

## 2 METHODS

### 2.1 Patients

In this retrospective cohort study, we analyzed adult patients who were consecutively prescribed a WCD in Germany from April 2015 to May 2018. Consent was obtained from all patients to use their data. Four weeks of physical activity beginning with the first week of WCD wear were used for the current study. To be included in the dataset, patients had to have a minimum threshold of at least 140 hours of WCD wear during the first week of WCD prescription. Patients were excluded if they died of unknown death over the prescribed wearing period, had incomplete data, or withdrawn WCD. Patients were included in the shocked group in they received an appropriate shock. Patients who received an inappropriate shock were included in the nonshocked group.

### 2.2 Device description

The WCD (LifeVest, ZOLL, Pittsburgh, PA) automatically detects and treats sustained VT and VF. The device contains a monitor (about 0.77 kg) worn on a hip holster (or shoulder strap) and an electrode belt; the latter holds 4 dry electrodes creating 2 surface electrocardiogram (ECG) channels, positioned in side‐to‐side (SS) and front‐to‐back (FB) configurations. A three‐axis accelerometer in the electrode belt determines the patient's step count and body position. The chest garment contains two defibrillation pads on the back and one at the left anterior part of the chest, close to the apical region of the heart. A pair of response buttons on the monitor allows a conscious patient to prevent a potential shock. A description of the WCD technical details have been reported elsewhere in the literature.^15^

### 2.3 Statistical analysis

Descriptive statistics were used to summarize demographic and shock data. Activity data, defined as step count, was continuously recorded for each patient during WCD wear. Average daily step count was calculated each week for 4 consecutive weeks. Data were excluded for one patient due to their Week 3 step count being an extreme outlier (*z*‐score = 13). Receiver operating characteristic (ROC) curves were constructed to determine the optimal cutoff for predicting group membership (shocked vs nonshocked) using step count during week 1. The Youdin index method was applied to preserve sensitivity and specificity when identifying the step count cutoff value. A *t*‐test was used to assess differences in physical activity between the shocked and nonshocked groups at weeks 2, 3, and 4. A *P*‐value < .05 was considered statistically significant. All analyses were performed using SPSS version 25 (IBM SPSS Statistics, IBM Corporation, Armonk, NY).

## 3 RESULTS

### 3.1 Patients

A total of 5528 patients were screened, 1471 patients were excluded for incomplete data (n = 97), not meeting minimum age (n = 15) or WCD hours of wear requirements (n = 677), withdrawn WCD (withdrawn could be due to several factors including: skin irritation, noncompliance, insurance reasons) (n = 630), or if they died of unknown death during the study period (n = 52). In the final sample, 4057 patients were included. Demographic variables for the full sample, and by group (shocked vs nonshocked) are summarized in Table 1. Eighty‐one patients (2%) received an appropriate shock for sustained VT/VF during WCD wear. On average, patients in the shocked group were older (64, SD = 14 years) than patients in the nonshocked group (60, SD = 13 years, *P* = .01). Of the 81 patients who received a shock, 9 (11%) did not survive the shock event.

**Table 1:** Baseline characteristics by group

	Total sample	Shocked	Nonshocked	*P*‐value
*N* (%)	4057	81 (2)	3976 (98)	
Age mean (SD)	60 (13)	64 (14)	60 (13)	.01
Male (%)	3239 (80)	67 (83)	3172 (80)	.51
Daily step count week 1 median (IQR)	4771 (2739‐7318)	2456 (961‐4185)	4821 (2779‐7350)	<.001
*WCD indication (%)*				<.001
Recent PCI	1182 (29.1)	23 (28.4)	1159 (29.1)	
Dilated cardiomyopathy	1007 (24.8)	8 (9.9)	999 (25.1)	
Other	952 (23.5)	25(30.9)	927 (23.3)	
NICM	364 (9.0)	4(4.9)	360 (9.1)	
Myocarditis	242 (6.0)	5(6.2)	237 (6.0)	
ICD explant	163 (4.0)	13(16.0)	150 (3.8)	
Recent CABG	147 (3.6)	3(3.7)	144 (3.6)	

ICD, implantable cardioverter defibrillator; IQR, interquartile range; NICM, nonischemic cardiomyopathy; PCI, percutaneous coronary intervention; SD, standard deviation; WCD, wearable cardioverter defibrillator.

### 3.2 Activity

During week 1, patients in the shocked group had significantly fewer steps compared to the not‐shocked group (Table 1).

Based on the ROC curve outcome, daily step count during week 1 demonstrated good accuracy in predicting group (shocked vs nonshocked (area under the curve, c‐index = .71, 95% CI = .65‐.77, *P* < .001). The Youdin index was applied and a cutoff daily step count of 3637 during week 1 was identified with maximum sensitivity and specificity, 73% and 65%, respectively. In our sample, 36% (n = 1463) of patients walked fewer than 3637 steps a day during week 1. These patients were five times more likely to experience a shock than patients with more than 3637 steps per day during this time period (OR = 4.91, 95% CI = 3.0‐8.05, *P* < .001).

**Figure 1:**
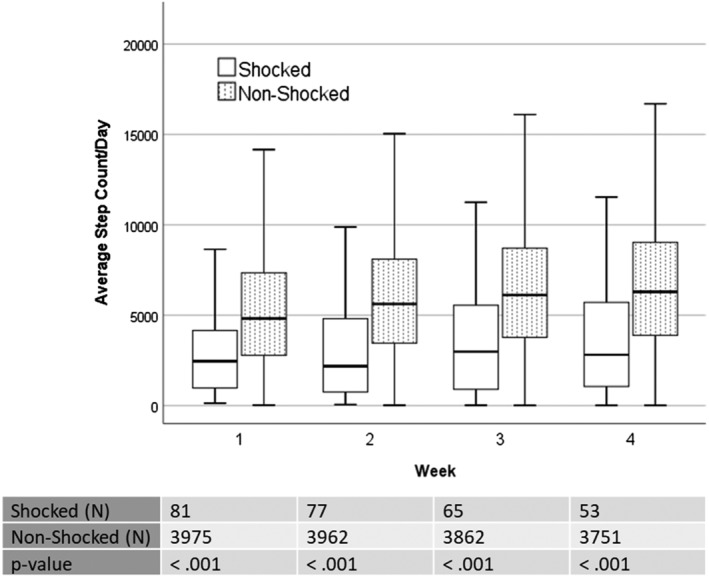
Daily average step count across the 4‐week study period. Step count was significantly lower in the appropriately shocked compared to nonshocked group across each of the 4 weeks

A logistic regression was performed to predict group membership from step count; age and WCD indication was univariately associated with the group and were therefore entered into the model as additional predictors. A binary variable was created for step count based on the cutoff of 3637 steps per day during week 1 (ie, greater than 3637 or less than 3637 steps). With age and WCD indication entered into the model, step count during week 1 remained a significant predictor of group. In this multivariate analysis, patients who walked fewer than 3637 steps per day during the first week of WCD prescription were 4.3 times more likely to experience a shock than those who walked more than 3637 steps per day (OR = 4.29, 95% CI = 2.58‐7.15, *P* < .001). Step count over the following 3 weeks remained significantly lower in the shocked group compared to the nonshocked group (Figure 1).

## 4 DISCUSSION

Less physical activity in the first week of WCD use is associated with shocked VT/VF events in the first month of WCD use. Patients who received a shock walked nearly half as many steps as patients in the nonshocked group. A cutoff value of 3637 steps during the first week of WCD use successfully identified shocked and nonshocked patients, even after controlling for differences in age between the two groups. Among patients who were shocked, 73% had fewer than 3637 steps during week 1.

Previous work has shown the utility of physical activity in predicting atrial arrhythmias, heart failure hospitalization, and death.^5^, ^6^, ^7^, ^8^, ^9^, ^10^ Results from the present study add to this literature by demonstrating the utility of accelerometer‐acquired activity in predicting sustained VT/VF in a population of WCD patients with a low ejection fraction. Furthermore, a clinically meaningful daily step count has been identified allowing clinicians to set a goal for their patients to reach a minimum step count of approximately 4000 steps each day to reduce the likelihood of sustained VT/VF.

Unlike many of the risk factors for sudden cardiac death, physical activity is a modifiable risk factor. Reductions in heart failure risk are seen among patients adhering to guideline‐recommended levels of activity.^16^ The recommended level of 150‐300 minutes of moderately intense activity per week may seem out of reach for many heart failure patients; however, even modest increases in physical activity can have beneficial cardiac outcomes.^17^ Studies of heart failure patients participating in short‐term cardiac rehabilitation programs have shown significant improvements in peak oxygen uptake and left ventricular ejection fraction.^18^, ^19^

The current study presents physical activity as a function of step count. Studies from North American and Australia report 20‐35% of a population‐based sample of adults own a wearable activity tracker.^20^, ^21^ The ubiquity of commercially available step count monitors makes this metric of activity more accessible for patients than the traditionally reported minutes spent active. A systematic review of 26 studies found activity tracker utilization was associated with significant improvements in physical activity among outpatient adults.^22^ Among post‐myocardial infarction patents, activity self‐monitoring was associated with exercise maintenance 6 months after completing a cardiac rehabilitation program compared to a randomized nonself‐monitoring group.^23^ These studies suggest objective monitoring of daily activity empowers patients to engage in their own activity rehabilitation process. Outcomes from the current study allow clinicians to encourage patients to set specific, evidence‐based activity goals and use activity trackers to monitor progress towards these goals.

The ubiquity of patient owned activity trackers and the increased available of cardiac devices with accelerometer recorded activity that can be provided to physicians increases the utility of activity metrics for monitoring cardiac care. Changes in activity may signal declining health and worsening heart failure. Accelerometer containing cardiac devices, including the WCD, can provide continuous real‐time activity monitoring of at‐risk patients. The present study suggests reduced activity may be a useful marker to identify patients at increased risk of sustained VT/VF, further adding to sudden cardiac death risk stratification beyond left ventricular ejection fraction. Others have proposed the presence of warning symptoms and signs, such as angina pectoris and dyspnea that may forecast sudden cardiac arrest.^24^, ^25^, ^26^ Identification of a decline in activity as a precedent to sustained VT/VF may allow physicians time to intervene and make pharmacological or other treatment adjustments.

### 4.1 Study limitations

The retrospective design of this study does not allow for a causal conclusion to be drawn between decreased activity and sustained VT/VF. It remains a possibility that patients who are less active are sicker and therefore at a greater risk of death. However, in the MADIT‐CRT, reduced activity during the week of a nonfatal ventricular arrhythmia was associated with an increased probability of death.^7^ These findings suggest a decline in physical activity may precede sustained VT/VF, and that decreased activity associated with VT/VF may determine the significance of the event for future outcomes, including death. Prospective studies would be helpful to determine the possible benefit of physical activity in preventing sudden cardiac death. In addition, randomization based on disease state would provide information about the relationship between change in physical activity and heart failure progression.

A second limitation of this study is the lack of co‐variates, including underlying heart disease, mobility restrictions, pharmacological management, and NYHA class, all of which may influence a proportion of the relationship found between decreased activity and increased incidence of shock. The study sample, although large in number, was pulled from a commercial database with limited co‐variate data available for reporting. Another limitation, similar to many other cardiac study populations, our sample was predominately male. A significant gender difference in rates of physical activity may exist. An international study of 3572 acute‐myocardial infarction (MI) patients found women were 37% more likely to exhibit low levels of activity compared to males.^27^ At 12 months post‐MI, only 36% of women compared to 48% of men met the recommended level of physical activity. Encouragingly, a meta‐analysis including 370 460 heart failure patients found the benefit of higher levels of physical activity in reducing heart failure risk was similar between men and women.^28^ Though men and women may benefit from physical activity, additional investigation is needed to determine if the cutoff values in activity detected from predominately male cardiac populations are applicable to female patients.

Engaging in regular physical activity is critical for optimal heart failure management. Patients who manifest spontaneous VT/VF have a lower average daily step count than those who do not have a sustained VT/VF event. Among patients at risk for sudden cardiac arrest, encouraging a minimum of 4000 steps per day could reduce the likelihood of sustained VT/VF.

## CONFLICT OF INTEREST

The authors declared the following potential conflicts of interest with respect to the research, authorship, and/or publication of this article: Ashley Burch has research grants from ZOLL Medical. These funds are directed to East Carolina University. Samuel Sears has research grants from Medtronic and ZOLL Medical. These funds are directed to East Carolina University. Samuel Sears serves as a consultant to Medtronic, Abbot/St Jude Medical, and ZOLL Medical. The other authors declare that they have no known conflicts of interest.
